# Supplementary material for: The Systems Biology Research Tool: evolvable open-source software
Source: BMC Syst Biol. 2008 Jun 29;2:55. doi: 10.1186/1752-0509-2-55 (PMC2446383; doi:10.1186/1752-0509-2-55)
Supplement: Additional file 1 — SBRT Archive. An archive of the current version of the Systems Biology Research Tool. [file 1752-0509-2-55-S1.zip › sbrt-1.4.0/doc/developers_guide/api/sbrt/kernel/fba/comp/AbstractFluxIntervalComputer.html]

AbstractFluxIntervalComputer


|  |  |  |
| --- | --- | --- |
| **Overview** \| **Package** \| **Class** \| **Use** \| **Tree** \| **Deprecated** \| **Index** \| **Help** | |  |
| **PREV CLASS**   **NEXT CLASS** | **FRAMES**    **NO FRAMES**     **All Classes** |  |
| SUMMARY: NESTED \| FIELD \| CONSTR \| METHOD | DETAIL: FIELD \| CONSTR \| METHOD |  |


---


## sbrt.kernel.fba.comp Class AbstractFluxIntervalComputer

```
java.lang.Object
  sbrt.kernel.fba.comp.AbstractCvComputer
      sbrt.kernel.fba.comp.AbstractFluxIntervalComputer
```

**All Implemented Interfaces:**: FluxCvComputer, FluxIntervalComputer

**Direct Known Subclasses:**: FastMsFluxRangeComputer, MsFluxIntervalComputer, ReverseRxnFluxIntervalComputer, SimpleFluxIntervalComputer

---

``` public abstract class AbstractFluxIntervalComputer extends AbstractCvComputer implements FluxIntervalComputer ```

This abstract class is a skeleton implemenation of
`FluxIntervalComputer`.

**Author:**
:   This class was written and documented by Jeremiah Wright
    while in the Wagner lab.

---

| **Field Summary** | |
| --- | --- |
| `static double` | `ZERO_CUTOFF`             The default zero cutoff. |


**Constructor Summary**

`AbstractFluxIntervalComputer(FbaOptComputer computer)`
  
          Constructs a new flux interval computer with the default zero cutoff.

`AbstractFluxIntervalComputer(FbaOptComputer computer,
double zeroCutoff)`
  
          Constructs a new flux interval computer with the provided zero cutoff.


| **Method Summary** | |
| --- | --- |
| `protected  double` | `getBoundedMax(MathExpr<java.lang.String> rxnNameExpr)`             Computes the maximum value of the provided objective function. |
| `protected  double` | `getBoundedMax(java.lang.String reactionName)`             Computes the maximum flux of the reaction with the provided name. |
| `protected  double` | `getBoundedMaxWithReverseConstrained(java.lang.String reactionName)`             Computes the maximum flux of the reaction with the provided name while constraining the fluxes of its reverse reactions to their minimum. |
| `protected  double` | `getBoundedMin(MathExpr<java.lang.String> rxnNameExpr)`             Computes the minimum value of the provided objective function. |
| `protected  double` | `getBoundedMin(java.lang.String reactionName)`             Computes the minimum flux of the reaction with the provided name. |
| `double` | `getConstraintTolerance()`             Returns the constraint tolerance used by this flux interval computer. |
| `Interval` | `getFluxConstraint(java.lang.String rxnName)`             Returns the current flux constraint on the reaction with the provided name. |
| `java.util.Map<java.lang.String,Interval>` | `getFluxConstraints()`             Returns all current flux constraints. |
| `protected  Interval` | `getInterval(java.lang.String rxn, Interval fluxConstraint, double computedMin, double computedMax)`             Returns the provided minimum and maximum fluxes as an interval. |
| `protected  java.util.Map<java.lang.String,Interval>` | `getIntervals(java.util.Map<java.lang.String,java.lang.Double> fluxMinima, java.util.Map<java.lang.String,java.lang.Double> fluxMaxima)`             Returns the provided minima and maxima as intervals. |
| `protected  double` | `getMaximum(MathExpr<java.lang.String> rxnNameExpr)`             Computes the maximum value of the provided objective function. |
| `protected  double` | `getMaximum(java.lang.String reactionName)`             Computes the maximum flux of the reaction with the provided name. |
| `protected  double` | `getMaxWithReverseConstrained(java.lang.String reactionName)`             Computes the maximum flux of the reaction with the provided name while constraining the fluxes of its reverse reactions to their minimum. |
| `protected  double` | `getMinimum(MathExpr<java.lang.String> rxnNameExpr)`             Computes the minimum value of the provided objective function. |
| `protected  double` | `getMinimum(java.lang.String reactionName)`             Computes the minimum flux of the reaction with the provided name. |
| `protected  java.util.Map<java.lang.String,java.util.Set<java.lang.String>>` | `getReverseRxnNames(java.util.Collection<java.lang.String> reactionNames)`             Returns the name of each provided reaction mapped to the names of each of its reverse reactions. |
| `Interval` | `getRxnExprConstraint(MathExpr<java.lang.String> rxnNameExp)`             Returns the current constraint on the provided expression. |
| `java.util.Map<MathExpr<java.lang.String>,Interval>` | `getRxnExprConstraints()`             Returns all current flux expression constraints. |
| `double` | `getZeroCutoff()`             Returns the zero cutoff used by this flux interval computer. |
| `void` | `restoreFluxConstraint(java.lang.String reactionName)`             Restores the flux constraint for the reaction with the provided name to the value contained in the fluxome. |
| `void` | `restoreFluxConstraints()`             Restores the flux constraints for all reactions in the fluxome to those values contained in the fluxome. |
| `void` | `restoreFluxConstraints(java.util.Collection<java.lang.String> reactionNames)`             Restores the flux constraints for the provided reaction names to the values contained in the fluxome. |
| `void` | `restoreRxnExprConstraint(MathExpr<java.lang.String> rxnNameExp)`             Restores the constraint on the provided flux expression to the value contained in the fluxome. |
| `void` | `restoreRxnExprConstraints()`             Restores the constraints on all flux expressions to the values contained in the fluxome. |
| `void` | `restoreRxnExprConstraints(java.util.Collection<? extends MathExpr<java.lang.String>> rxnNameExps)`             Restores the constraints on the provided expressions to the values contained in the fluxome. |
| `void` | `setFluxConstraint(java.lang.String reactionName, Interval c)`             Changes the flux constraint of the reaction with the provided name. |
| `void` | `setFluxConstraints(java.util.Map<java.lang.String,Interval> fluxConstraints)`             Changes the flux constraints on more than one reaction. |
| `void` | `setRxnExprConstraint(MathExpr<java.lang.String> e, Interval c)`             Changes the constraint on the provided mathematical expression of fluxes. |
| `void` | `setRxnExprConstraints(java.util.Map<? extends MathExpr<java.lang.String>,Interval> constraints)`             Changes the constraints on more than one mathematical expression of fluxes. |

**Methods inherited from class sbrt.kernel.fba.comp.AbstractCvComputer**

`checkFluxConstraint, checkNumber, checkRxnExpr, checkRxnExprConstraint, checkRxnName, checkRxnNames, getFluxome, getRxnExpr, getRxnExpr`

**Methods inherited from class java.lang.Object**

`clone, equals, finalize, getClass, hashCode, notify, notifyAll, toString, wait, wait, wait`

**Methods inherited from interface sbrt.kernel.fba.comp.FluxIntervalComputer**

`getIntervals, getIntervals, getProgress`

**Methods inherited from interface sbrt.kernel.fba.comp.FluxCvComputer**

`getFluxome`

**Field Detail**

### ZERO\_CUTOFF

```
public static final double ZERO_CUTOFF
```

:   The default zero cutoff.

    **See Also:**: Constant Field Values


**Constructor Detail**

### AbstractFluxIntervalComputer

```
public AbstractFluxIntervalComputer(FbaOptComputer computer)
```

:   Constructs a new flux interval computer with the default zero cutoff.

    **Parameters:**: `computer` - the computer with which all optimizations will be performed. **See Also:**: `ZERO_CUTOFF`

---


### AbstractFluxIntervalComputer

```
public AbstractFluxIntervalComputer(FbaOptComputer computer,
                                    double zeroCutoff)
```

:   Constructs a new flux interval computer with the provided zero cutoff.

    **Parameters:**: `computer` - the computer with which all optimizations will be performed.: `zeroCutoff` - the zero cutoff to be used by this computer.


**Method Detail**

### getConstraintTolerance

```
public double getConstraintTolerance()
```

:   Returns the constraint tolerance used by this flux interval computer.

    :   **Specified by:**: `getConstraintTolerance` in interface `FluxIntervalComputer`
    :   **Returns:**: the constraint tolerance used by this flux interval computer.

---


### getZeroCutoff

```
public double getZeroCutoff()
```

:   Returns the zero cutoff used by this flux interval computer.

    :   **Specified by:**: `getZeroCutoff` in interface `FluxIntervalComputer`
    :   **Returns:**: the zero cutoff used by this flux interval computer.

---


### getReverseRxnNames

```
protected java.util.Map<java.lang.String,java.util.Set<java.lang.String>> getReverseRxnNames(java.util.Collection<java.lang.String> reactionNames)
```

:   Returns the name of each provided reaction mapped to the names of
    each of its reverse reactions.

    :   **Parameters:**: `reactionNames` - the names of the reactions whose reverse reactions are to be determined. **Returns:**: reaction names mapped to the set of names of their reverse reactions.

---


### getMaximum

```
protected double getMaximum(java.lang.String reactionName)
```

:   Computes the maximum flux of the reaction with the provided name.

    :   **Parameters:**: `reactionName` - the name of the reaction whose maximum flux will be computed. **Returns:**: the maximum flux of the reaction with the provided name, or Double.POSITIVE\_INFINITY if the flux is unbounded. **Throws:**: `FbaOptException` - if an optimal solution was not found.: `java.lang.IllegalArgumentException` - if the provided string is not a reaction name of the fluxome used by this flux interval computer.

---


### getMaximum

```
protected double getMaximum(MathExpr<java.lang.String> rxnNameExpr)
```

:   Computes the maximum value of the provided objective function.

    :   **Parameters:**: `rxnNameExpr` - a mathematical expression whose variables are reaction names. **Returns:**: the maximum value of the provided objective function, or Double.POSITIVE\_INFINITY if the problem is unbounded. **Throws:**: `FbaOptException` - if an optimal solution was not found.: `java.lang.IllegalArgumentException` - if the provided expression contains a variable that is not a reaction name.: `java.lang.NullPointerException` - if the provided expression is `null`.

---


### getMinimum

```
protected double getMinimum(java.lang.String reactionName)
```

:   Computes the minimum flux of the reaction with the provided name.

    :   **Parameters:**: `reactionName` - the name of the reaction whose minimum flux will be computed. **Returns:**: the minimum flux of the reaction with the provided name, or Double.NEGATIVE\_INFINITY if the flux is unbounded. **Throws:**: `FbaOptException` - if an optimal solution was not found.: `java.lang.IllegalArgumentException` - if the provided string is not a reaction name of the fluxome used by this flux interval computer.

---


### getMinimum

```
protected double getMinimum(MathExpr<java.lang.String> rxnNameExpr)
```

:   Computes the minimum value of the provided objective function.

    :   **Parameters:**: `rxnNameExpr` - a mathematical expression whose variables are reaction names. **Returns:**: the minimum value of the provided objective function, or Double.NEGATIVE\_INFINITY if the problem is unbounded. **Throws:**: `FbaOptException` - if an optimal solution was not found.: `java.lang.IllegalArgumentException` - if the provided expression contains a variable that is not a reaction name.: `java.lang.NullPointerException` - if the provided expression is `null`.

---


### getBoundedMin

```
protected double getBoundedMin(MathExpr<java.lang.String> rxnNameExpr)
```

:   Computes the minimum value of the provided objective function.

    :   **Parameters:**: `rxnNameExpr` - a mathematical expression of reaction names. **Returns:**: the minimum value of the provided objective function. **Throws:**: `UnboundedVariableException` - if the optimization problem proves to be unbounded.

---


### getBoundedMax

```
protected double getBoundedMax(MathExpr<java.lang.String> rxnNameExpr)
```

:   Computes the maximum value of the provided objective function.

    :   **Parameters:**: `rxnNameExpr` - a mathematical expression of reaction names. **Returns:**: the maximum value of the provided objective function. **Throws:**: `UnboundedVariableException` - if the optimization problem proves to be unbounded.

---


### getBoundedMin

```
protected double getBoundedMin(java.lang.String reactionName)
```

:   Computes the minimum flux of the reaction with the provided name.

    :   **Parameters:**: `reactionName` - the name of a reaction in the fluxome used by this computer. **Returns:**: the minimum flux of the reaction with the provided name. **Throws:**: `UnboundedVariableException` - if the optimization problem proves to be unbounded.

---


### getBoundedMax

```
protected double getBoundedMax(java.lang.String reactionName)
```

:   Computes the maximum flux of the reaction with the provided name.

    :   **Parameters:**: `reactionName` - the name of a reaction in the fluxome used by this computer. **Returns:**: the maximum flux of the reaction with the provided name. **Throws:**: `UnboundedVariableException` - if the optimization problem proves to be unbounded.

---


### getBoundedMaxWithReverseConstrained

```
protected double getBoundedMaxWithReverseConstrained(java.lang.String reactionName)
```

:   Computes the maximum flux of the reaction with the provided name while
    constraining the fluxes of its reverse reactions to their minimum.

    :   **Parameters:**: `reactionName` - the name of a reaction in the fluxome used by this computer. **Returns:**: the maximum flux of the reaction with the provided name. **Throws:**: `UnboundedVariableException` - if the optimization problem proves to be unbounded.

---


### getMaxWithReverseConstrained

```
protected double getMaxWithReverseConstrained(java.lang.String reactionName)
```

:   Computes the maximum flux of the reaction with the provided name while
    constraining the fluxes of its reverse reactions to their minimum.

    :   **Parameters:**: `reactionName` - the name of a reaction in the fluxome used by this computer. **Returns:**: the maximum flux of the reaction with the provided name, or Double.POSITIVE\_INFINITY if the problem is unbounded.

---


### getInterval

```
protected Interval getInterval(java.lang.String rxn,
                               Interval fluxConstraint,
                               double computedMin,
                               double computedMax)
```

:   Returns the provided minimum and maximum fluxes as an interval. This
    method performs several error-checking procedures to ensure the provided
    values are consistent with each other and with the defined constraint
    tolerance and zero cutoff. It also corrects for small numerical errors in
    the provided computed values.

    :   **Parameters:**: `rxn` - the name of the reaction whose lower and upper flux bounds have been computed.: `fluxConstraint` - the current constraint on the flux whose lower and upper bounds have been computed.: `computedMin` - the computed lower flux bound.: `computedMax` - the computed upper flux bound. **Returns:**: the provided minimum and maximum fluxes as an interval. **Throws:**: `ConstraintViolationException` - if either of the computed fluxes lie outside the provided flux constraint by more than the defined constraint tolerance.: `IllegalBoundsException` - if the provided minimum is greater than the provided maximum by more than the defined zero cutoff.

---


### getIntervals

```
protected java.util.Map<java.lang.String,Interval> getIntervals(java.util.Map<java.lang.String,java.lang.Double> fluxMinima,
                                                                java.util.Map<java.lang.String,java.lang.Double> fluxMaxima)
```

:   Returns the provided minima and maxima as intervals. This method
    performs several error-checking procedures to ensure the provided
    minima and maxima are consistent with each other and with the defined
    constraint tolerance and zero cutoff. It also corrects for small
    numerical errors in the provided computed values.

    :   **Parameters:**: `fluxMinima` - the computed flux minima.: `fluxMaxima` - the computed flux maxima. **Returns:**: reaction names mapped to the interval dictated by the provided minimum and maximum values.

---


### setFluxConstraint

```
public void setFluxConstraint(java.lang.String reactionName,
                              Interval c)
```

:   Changes the flux constraint of the reaction with the provided name.

    :   **Specified by:**: `setFluxConstraint` in interface `FluxCvComputer`
    :   **Parameters:**: `reactionName` - the name of the reaction whose flux constraint is to be changed.: `c` - the new constraint.

---


### setFluxConstraints

```
public void setFluxConstraints(java.util.Map<java.lang.String,Interval> fluxConstraints)
```

:   Changes the flux constraints on more than one reaction.

    :   **Specified by:**: `setFluxConstraints` in interface `FluxCvComputer` **Overrides:**: `setFluxConstraints` in class `AbstractCvComputer`
    :   **Parameters:**: `fluxConstraints` - reaction names mapped to their respective flux constraints.

---


### setRxnExprConstraint

```
public void setRxnExprConstraint(MathExpr<java.lang.String> e,
                                 Interval c)
```

:   Changes the constraint on the provided mathematical expression
    of fluxes.

    :   **Specified by:**: `setRxnExprConstraint` in interface `FluxCvComputer`
    :   **Parameters:**: `e` - a matheamtical expression of reaction names.: `c` - the new constraint.

---


### setRxnExprConstraints

```
public void setRxnExprConstraints(java.util.Map<? extends MathExpr<java.lang.String>,Interval> constraints)
```

:   Changes the constraints on more than one mathematical expression
    of fluxes.

    :   **Specified by:**: `setRxnExprConstraints` in interface `FluxCvComputer` **Overrides:**: `setRxnExprConstraints` in class `AbstractCvComputer`
    :   **Parameters:**: `constraints` - mathematical expressions of reaction names mapped to their new respective constraints.

---


### restoreFluxConstraint

```
public void restoreFluxConstraint(java.lang.String reactionName)
```

:   Restores the flux constraint for the reaction with the provided
    name to the value contained in the fluxome.

    :   **Specified by:**: `restoreFluxConstraint` in interface `FluxCvComputer`
    :   **Parameters:**: `reactionName` - the name of the reaction whose flux constraint is to be restored.

---


### restoreFluxConstraints

```
public void restoreFluxConstraints(java.util.Collection<java.lang.String> reactionNames)
```

:   Restores the flux constraints for the provided reaction names to the
    values contained in the fluxome.

    :   **Specified by:**: `restoreFluxConstraints` in interface `FluxCvComputer` **Overrides:**: `restoreFluxConstraints` in class `AbstractCvComputer`
    :   **Parameters:**: `reactionNames` - the reaction names whose flux constraints are to be restored.

---


### restoreFluxConstraints

```
public void restoreFluxConstraints()
```

:   Restores the flux constraints for all reactions in the fluxome to those
    values contained in the fluxome.

    :   **Specified by:**: `restoreFluxConstraints` in interface `FluxCvComputer` **Overrides:**: `restoreFluxConstraints` in class `AbstractCvComputer`

---


### restoreRxnExprConstraint

```
public void restoreRxnExprConstraint(MathExpr<java.lang.String> rxnNameExp)
```

:   Restores the constraint on the provided flux expression to the value
    contained in the fluxome.

    :   **Specified by:**: `restoreRxnExprConstraint` in interface `FluxCvComputer`
    :   **Parameters:**: `rxnNameExp` - the expression of reaction names whose contraint is to be restored.

---


### restoreRxnExprConstraints

```
public void restoreRxnExprConstraints(java.util.Collection<? extends MathExpr<java.lang.String>> rxnNameExps)
```

:   Restores the constraints on the provided expressions to the values
    contained in the fluxome.

    :   **Specified by:**: `restoreRxnExprConstraints` in interface `FluxCvComputer` **Overrides:**: `restoreRxnExprConstraints` in class `AbstractCvComputer`
    :   **Parameters:**: `rxnNameExps` - the reaction name expressions whose contraints are to be restored.

---


### restoreRxnExprConstraints

```
public void restoreRxnExprConstraints()
```

:   Restores the constraints on all flux expressions to the values
    contained in the fluxome.

    :   **Specified by:**: `restoreRxnExprConstraints` in interface `FluxCvComputer`

---


### getRxnExprConstraints

```
public java.util.Map<MathExpr<java.lang.String>,Interval> getRxnExprConstraints()
```

:   Returns all current flux expression constraints.

    :   **Specified by:**: `getRxnExprConstraints` in interface `FluxCvComputer`
    :   **Returns:**: mathematical expressions of reaction names mapped to their respective constraint.

---


### getFluxConstraints

```
public java.util.Map<java.lang.String,Interval> getFluxConstraints()
```

:   Returns all current flux constraints.

    :   **Specified by:**: `getFluxConstraints` in interface `FluxCvComputer`
    :   **Returns:**: reaction names mapped to their respective constraint.

---


### getRxnExprConstraint

```
public Interval getRxnExprConstraint(MathExpr<java.lang.String> rxnNameExp)
```

:   Returns the current constraint on the provided expression.

    :   **Specified by:**: `getRxnExprConstraint` in interface `FluxCvComputer`
    :   **Parameters:**: `rxnNameExp` - the mathematical expression of reaction names whose constraint is to be returned. **Returns:**: the current constraint on the provided expression.

---


### getFluxConstraint

```
public Interval getFluxConstraint(java.lang.String rxnName)
```

:   Returns the current flux constraint on the reaction with the provided
    name.

    :   **Specified by:**: `getFluxConstraint` in interface `FluxCvComputer`
    :   **Parameters:**: `rxnName` - the name of the reaction whose flux constraint is to be returned. **Returns:**: the current flux constraint on the reaction with the provided name.


---


|  |  |  |
| --- | --- | --- |
| **Overview** \| **Package** \| **Class** \| **Use** \| **Tree** \| **Deprecated** \| **Index** \| **Help** | |  |
| **PREV CLASS**   **NEXT CLASS** | **FRAMES**    **NO FRAMES**     **All Classes** |  |
| SUMMARY: NESTED \| FIELD \| CONSTR \| METHOD | DETAIL: FIELD \| CONSTR \| METHOD |  |


---
